# Exploratory analysis of multi‐trait coadaptations in light of population history

**DOI:** 10.1002/ece3.8755

**Published:** 2022-03-18

**Authors:** Reiichiro Nakamichi, Shuichi Kitada, Hirohisa Kishino

**Affiliations:** ^1^ 13519 Japan Fisheries Research and Education Agency Yokohama Japan; ^2^ Tokyo University of Marine Science and Technology Tokyo Japan; ^3^ Graduate School of Agriculture and Life Sciences The University of Tokyo Tokyo Japan; ^4^ The Research Institute of Evolutionary Biology Tokyo Japan; ^5^ AI/Data Science Social Implementation Laboratory Chuo University Tokyo Japan

**Keywords:** admixture graph, coadaptation of interacting traits, exploratory analysis, history of range expansion, polygenic traits, population structure

## Abstract

During the process of range expansion, populations encounter a variety of environments. They respond to the local environments by modifying their mutually interacting traits. Common approaches of landscape analysis include first focusing on the genes that undergo diversifying selection or directional selection in response to environmental variation. To understand the whole history of populations, it is ideal to capture the history of their range expansion with reference to the series of surrounding environments and to infer the multitrait coadaptation. To this end, we propose a complementary approach; it is an exploratory analysis using up‐to‐date methods that integrate population genetic features and features of selection on multiple traits. First, we conduct correspondence analysis of site frequency spectra, traits, and environments with auxiliary information of population‐specific fixation index (*F*_ST_). This visualizes the structure and the ages of populations and helps infer the history of range expansion, encountered environmental changes, and selection on multiple traits. Next, we further investigate the inferred history using an admixture graph that describes the population split and admixture. Finally, principal component analysis of the selection on edge‐by‐trait (SET) matrix identifies multitrait coadaptation and the associated edges of the admixture graph. We introduce a newly defined factor loadings of environmental variables in order to identify the environmental factors that caused the coadaptation. A numerical simulation of one‐dimensional stepping‐stone population expansion showed that the exploratory analysis reconstructed the pattern of the environmental selection that was missed by analysis of individual traits. Analysis of a public dataset of natural populations of black cottonwood in northwestern America identified the first principal component (PC) coadaptation of photosynthesis‐ vs growth‐related traits responding to the geographical clines of temperature and day length. The second PC coadaptation of volume‐related traits suggested that soil condition was a limiting factor for aboveground environmental selection.

## Graphical abstract

During the process of range expansion, populations encounter a variety of environments and respond to the local environments by modifying their mutually interacting traits. To capture the history of their range expansion with reference to the series of surrounding environments and to infer the multitrait coadaptation, we propose a complementary approach; it is an exploratory analysis using up‐to‐date methods that integrates population genetic features and features of selection on multiple traits.

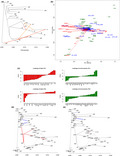

## 1 INTRODUCTION

Populations adapt to new environments through selection on preexisting alleles and/or new mutations in adaptation‐related loci in the genomes (Barrett & Schluter, 2008). Therefore, adaptation of populations of a species to novel environments changes allele frequencies of loci under selection. Environmental adaptation processes can also create significant differences in phenotypes and traits among populations of a species. When correlated with variation in environmental factors over local subpopulations (hereafter, populations), such variation in traits and phenotypes may reflect phenotypic plasticity or genetic adaptation of the populations. Coop et al. (2010) proposed that significant correlations could be detected between single nucleotide polymorphism (SNP) allele frequencies and environmental variables, bypassing trait variables. Through the annotation of identified SNPs, it may be possible to characterize the type of adaptation.

Adaptation to environmental factors can change traits and phenotypes of a species, thereby creating population structure underpinned by functional loci. Geographical isolation, which can lead to reproductive isolation and consequent differences in allele frequencies of neutral loci, also contributes to population structuring (Wright, 1965). Divergent selection in an environmental gradient may affect genome‐wide population structure (Nosil et al., 2009; Orsini et al., 2013). Empirical studies showed that aridity gradients caused geographically structured populations of Poaceae characterized by cytotype segregation of diploids and allotetraploids (Manzaneda et al., 2012). Geographic distance and habitat differences between populations impacted population structure of marine species (Bradbury & Bentzen, 2007; Jorde et al., 2015; Kitada et al., 2017). Therefore, population structure needs to be considered when analyzing correlations among genes, traits, and environmental factors across population samples taken from a wide range of geographical regions.

To identify genetic adaptations, the common landscape genomics approach identifies SNPs that are diversified among populations. Adaptations to a specific environment may be determined by identifying among‐population variation of allele frequencies that are associated with the variation of environmental values. Genome‐wide association study (GWAS) results and annotation are used to characterize the environmental adaptation of SNPs. Another approach is to infer the positive selection on a trait during its evolutionary history by estimating the change in allele frequencies of associated SNPs beyond the level of genetic drift. So‐called “genome scan methods” consider geographically structured populations and detect SNPs related to environmental variables, traits, and phenotypes (De Mita et al., 2013; De Villemereuil et al., 2014). For example, BayeScan (Foll & Gaggiotti, 2008) measures the significance of an SNP’s locus‐specific global *F*_ST_ value, which represents the amount of genetic variation among populations, in a Bayesian framework. Genotype–environment associations analyze the allele frequencies of SNPs in sampling locations and test their associations with the environmental variables (Capblancq et al., 2020). Bayenv (Coop et al., 2010) and the latent factor mixed model (Frichot et al., 2013) can detect SNPs that are highly correlated with environmental factors and traits on the basis of allele frequencies. Notably, these methods essentially do not require phenotypic data. Hence, they are valid, especially when the history is complex and cannot be appropriately measured by a few trait variables or the environmental selection on the phenotypes are not characterized (Capblancq et al., 2020). During the evolutionary history of range expansion, the frequencies of existing and derived alleles in a population vary stochastically, and various pressures of environmental selection affect the allele frequencies of related genes and phenotypes. Systematic information on the associations between traits and SNPs in some species, such as humans (Watanabe et al., 2019) and *Arabidopsis* (Togninalli et al., 2019), enabled mapping of adaptive evolution of polygenic traits on admixture graphs (Racimo et al., 2018).

However, wild populations change their distributions gradually or abruptly over generations and encounter a variety of environments. They adapt to the local environments by modifying, in balance, multiple traits that are mutually interrelated. In Bristol Bay, early spawners of sockeye salmon lay eggs in late July to mid‐August in small streams that have cold temperatures until hatching but spawners in late August to October in large rivers/lakes that experience much warmer temperature, where the average egg size also reflects the habitat‐specific incubation environment and tradeoffs between egg size and number of eggs are population‐specific (Hilborn et al., 2003). Such reproductive traits adapted to specific environment might be controlled by related genes. Gonadotropin‐releasing hormone increases in adult salmon brains during homing migration and controls gonadal maturation during the final phases of upstream migration to spawn (Ueda, 2019). Moreover, populations of walking stick insects diverged in body size, shape, host preference, and behavior in parallel with the divergence of their host plant species (Nosil et al., 2002). Many plant species experience regular wildfire and have evolved sets of traits to adapt to this natural selection pressure. By using phylogenetic independent contrasts, Schwilk and Ackerly (2001) tested whether pines improved their traits for a survival/avoidance strategy or a fire‐embracing strategy. Phylogenetic analysis of 38 pine species demonstrated that much variation in trait evolution occurred along a fire‐surviving/fire‐embracing axis.

Here, we propose a complementary approach to identify multitrait coadaptation to a set of environmental changes that populations encountered in the history of their geographical distribution. This is an exploratory analysis that integrates features of population genetic information and trait‐specific positive selection. These features are generated by up‐to‐date inference methods. Correspondence analysis visualizes the population structure with reference to the traits and environmental variables; the colors of the populations represent their ages and help infer their history of range expansion, the environmental selection pressures on the populations, and change in their traits in response. To characterize the environmental selection on the traits, we conducted principal component analysis (PCA) on a selection on edge‐by‐trait (SET) matrix. The biplot identifies the principal multitrait coadaptation, and the relevant edges of the admixture graph describe the history of population splits and admixture. To identify the environmental factors that caused the coadaptation, we newly define the factor loadings of the environmental variables based on the factor loadings of the traits and the current among‐population correlations, which are overlaid on the biplot. Once we gain insight into the pattern of multitrait coadaptation and their driving force of environmental factors, we in turn confirm the SNPs controlling the traits to examine the genetic changes that enabled the adaptations. The R codes for our representation method and simulations of population colonization used in this study are available in the Supporting Information. This approach also accepts SNP genotype data and reads Genepop format (Raymond & Rousset, 1995; Rousset, 2008).

## 2 MATERIALS AND METHODS

### 2.1 Correspondence analysis: chronologically ordered population structure in the map of traits and environmental variables

To first gain insight into the history of a population's range expansion and the environmental adaptation of their traits, we applied correspondence analysis to the data of the SNP allele frequencies, the environmental variables, and the mean values of the traits in each population. It characterizes the populations based on genetic, environmental, and trait variables together. Since SNP site frequencies comprise the majority of the data, the locations of the populations in the plot of correspondence analysis reflect the population structure. It provides a cue on the traits and environmental variables, and SNPs (if any), which are unique to some populations. By adding a crude measure of their ages as below, the plot provides a crude sketch on the history of range expansion of the populations, the environmental change they experienced and the associated traits adaptation. For SNP allele frequencies, we used the frequencies of derived alleles to infer signatures of environmental adaptation. However, it is difficult to know which allele is derived in data without information on the states in closely related species. In this paper, we adopted an ad hoc approach of using the frequencies of minor alleles as a substitute and expected that the frequencies of most derived alleles would still be low. This simple assignment of derived alleles may have errors, but we hoped that it would capture the SNPs with enhanced allele frequencies that facilitated adaptation to the local environments. To distinguish the association on the basis of positive and negative correlations, we introduced two types of environmental variables: the original environmental value itself and the sign‐reversed value of the original value. Genes and traits that had a positive/negative correlation with the original environmental factors were connected to the original/sign‐reversed environmental variables.

To understand the populations in the context of the evolutionary change across their distributional range, we assigned a gradient of colors to the populations. The colors represent population‐specific fixation index (*F*_ST_) (Weir & Goudet, 2017). Population‐specific *F*_ST_ estimates the genetic deviation from the ancestral population on the basis of the difference between the heterozygosity of all population pairs and the heterozygosity of each population. The Weir & Goudet population‐specific *F*_ST_ moment estimator can identify the source population and trace the history of range expansion based on heterozygosity under the assumption that populations closest to the ancestral population have the highest heterozygosity (Kitada et al., 2021). We extended the population‐specific *F*_ST_ estimator to overall loci as

$$\mathrm{ps}{\hat{F}}_{\mathrm{ST}}^{i}=\frac{\sum_{l=1}^{L}{(\tilde{M}}_{W,l}^{i}‐{\tilde{M}}_{l}^{B})}{\sum_{l=1}^{L}\left(1‐{\tilde{M}}_{l}^{B}\right)}\;,$$

where ${\tilde{M}}_{W,l}^{i}$ is the unbiased within‐population matching of two distinct alleles of locus l (l=1∼L) in population i (i=1∼K), and ${\tilde{M}}_{l}^{B}$ is the between‐population‐pair matching average over population pairs (Buckleton et al., 2016). To interpret the adaptation of the populations during their range expansion, we identified the significant correlations between the genes and the environmental variables (Appendix S1).

### 2.2 PCA of SET matrix: scores of edges of the admixture graph, and loadings of traits and environments

Visual inspection of multitrait coadaptation of the populations was investigated further using the admixture graph that describes the history of population splits and admixture. We conducted PCA of the SET matrix, which is explained below.

The dynamics of geographical distribution of populations are first approximated by two types of events, population differentiation and migration. Given the allele frequencies of the neutral SNP loci, TreeMix (Pickrell & Pritchard, 2012) estimates the admixture graph in the Bayesian framework. If there is no migration event, the graph is a bifurcating tree. The edges represent the genetic drifts from the ancestral population to the offspring population. With migration, the graph includes edges representing admixtures from source populations. PolyGraph (Racimo et al., 2018) identifies the positive selection on a trait occurring along the edges of the admixture graph as the change of the allele frequencies of SNPs associated with the trait beyond the level of genetic drift. The directional changes of their allele frequencies toward the increase/decrease of the trait values are called positive selection parameters.

To obtain the input data required for PolyGraph, we conducted GWAS for each of the traits considered (see Appendix S2). To understand the multitrait coadaptation, we made a SET matrix by binding the vectors listing the positive selection parameter values for the traits. Then, we performed PCA of the SET matrix. The vector of positive selection parameters that characterize the predicted increase/decrease of each trait value on the admixture graph was estimated using PolyGraph. To understand the environmental selection behind the coadaptation, we calculated the loadings of the environmental variables for each principal component (PC). The conditional loading of an environmental variable via a trait can be obtained as the product of the loading of the trait and the among‐population correlation between the trait and the environmental variable. By summing all conditional loadings of an environmental variable via each of the traits, we obtained the unconditioned loading of the environmental variable. To investigate the timing and geographical locations of the selections, we produced a colored admixture graph that emphasized the edges that experienced positive selections using TreeMix. For each principal component, the scores of the edges, obtained as a linear combination of the trait‐specific selection parameter values with the weight of the factor loadings, were mapped on the admixture graph. Red/blue colors on the edges of the admixture graph represented the selection toward the increase/decrease, respectively, of the “principal component trait” values.

### 2.3 Simulation of range expansion and adaptation

To illustrate how the overview generated by the exploratory analysis can be interpreted, we conducted a simulation of a simple scenario that mimics the colonization and environmental adaptation of *K* (= 25) linearly arrayed populations, Pop1, …, Pop25 (Austerlitz et al., 1997). The ancestral population, Pop1, accommodated an ancestral population of $N_{e}=10^{5}$. Once in 10 generations, habitat expansion occurred, and 1% of $N_{e}$ emigrated to the adjacent vacant population and increased the population size to the capacity $N_{e}=10^{4}$ in one generation (Figure S1). The allele frequencies of the SNPs in each population varied stochastically by genetic drift, colonization of its ancestral population, and selection pressure of the environments. We introduced two types of environmental factors, E_1_ and E_2_. E_1_ mimics the selection of a local environment, taking the value of 1 for six populations, Pop5, …, Pop10, and the value of 0 for the other populations. E_2_ mimics the selection of an environment associated with geographical cline, taking the value of 0 for Pop1, …, Pop14, followed by a linearly increase up to the value of 1 for Pop15, …, Pop25. Each of the environmental factors affected the survival of 15 traits. The ancestral nodes of Pop1, …, Pop25 were labeled as a1, …, a25, respectively.

Individuals in the populations had 15 traits, T1.1, …, T1.15, that were under the selection of E_1_, and 15 traits, T2.1, …, T2.15, that were under the selection of E_2_. The values of the traits of each type of environmental factor were not the result of pleiotropy of shared genomic loci but experienced the same selection pressure. In this study, we needed to simulate the dynamics of phenotypic traits and the allele frequencies at the loci that contribute to the traits. It was not computationally practical to specify the selection coefficients on the loci directly and to carry out individual‐based simulation. As a result, we simulated the dynamics of the population allele frequencies and mean traits. Each trait was polygenic, generated by summing 10 latent traits that are monogenic and affected by the environment. Adaptation to an environmental stress is often accompanied by the cost of reduced activity in the normal environment (e.g., Baucom & Mauricio, 2004); therefore, the derived alleles can adapt to the severe environment at the expense of cost in the normal environment. Our simulated derived adaptive allele at the genetic locus contributing to each latent trait had the selection coefficient of s=0.01 in the environment E = 1 and $\frac{1}{1+s}‐1=‐0.01$ in the environment E = 0 (see equations A4 and A5 in Appendix S3). Although the preexisting alleles and de novo mutations contribute to environmental adaptations, in this simulation, for simplicity we assumed that the relevant preexisting loci were already monomorphic in the ancestral population.

For the ancestral population, the allele frequencies at polymorphic loci were set to the theoretical equilibrium distribution, $f\left(q\right)\propto q^{‐1}{\left(1‐q\right)}^{‐1}$ (Wright, 1931). In the history of population expansion, populations randomly gave birth to neutral and adaptive mutations. The current genetic diversity reflects the genetic drift of polymorphic loci in the ancestral populations and that of de novo mutations that occurred in the history of the populations. For computational reasons, the initial frequency of the derived alleles at the newly generated loci was set to 0.01 in the populations where mutations occurred and 0 in the other populations; they mimicked new mutations that survived the initial phase after their birth. Population allele frequencies varied with random drift under a binomial distribution.

Neutral mutations were generated at random over generations and populations. A few adaptive mutations were generated every generation. Although many of these generated polymorphic loci became monomorphic before the present, which is the 260th generation, we kept loci that retained their polymorphism to this time. As a simplified procedure that mimics the SNP discovery process, we randomly selected a prespecified number of SNPs. In this simulation, we selected observed SNPs of 10,000 initial neutral loci, 500 newly derived neutral loci, and one newly derived environmentally adaptive locus for each latent trait. Then, we calculated the allele frequencies of the sample, which consisted of 50 individuals from each population.

### 2.4 Application to natural populations of black cottonwood data in North America

As an empirical example, we analyzed publicly available data that included genetic and trait information of 441 individuals of from natural populations of black cottonwood (*Populus trichocarpa*), which were collected from various regions over a range of 2500 km near the Canadian–US border at a latitude of 44′–59′ N, a longitude of 121′–138′ W, and an altitude of 0–800 m (Geraldes et al., 2013; McKown, Guy, Klápště, et al., 2014; McKown, Guy, Quamme, et al., 2014). The data included geographical information of sampling locations, genotypes of 34,131 SNPs (3516 genes), and values of stomatal anatomy, leaf tannin, ecophysiology, morphology, and disease. These individuals were geographically grouped into 25 drainages (populations) (Geraldes et al., 2014): 9 in northern British Colombia (NBC), 12 in southern British Colombia (SBC), 2 in inland British Colombia (IBC), and 2 in Oregon (ORE). Wild trees were replanted and grown in a common garden at British Columbia University. Common garden experiments control for the effects of phenotypic plasticity and, to a certain extent, genotype‐by‐environment interactions by growing individuals from different populations in a common environment (de Villemereuil et al., 2016).

By applying the method of landscape genomics, Geraldes et al. (2014) observed the pattern of isolation by distance. They found that genes involved in circadian rhythm and response to red/far‐red light are under diversifying selection for local adaptation. In the subarea of SBC, heat‐response genes were under diversifying selection. To characterize this selection, the association was determined between the allele frequencies of these genes and the geoclimatic variables. McKown, Guy, Klápště, et al. (2014) performed GWAS with 40 biomass, ecophysiology, and phenology traits, and found 410 significant SNPs. Further, they investigated the trade‐off in the adaptive change of stomatal density. Increased stomatal density enables efficient photosynthesis. However, it also increases the risk of pathogen invasion. They identified the genes controlling the stomatal traits and related their allele frequencies to geoclimatic variables.

In this paper, our exploratory approach directly searched for the major multitrait coadaptations to environments in light of population history. First, based on the allele frequencies of SNPs, and values of traits and environments, we conducted correspondence analysis that captured the population structure on a map of traits and environments. As a crude guide to the chronological order of the populations, populations were colored based on the population‐specific *F*_ST_ value. To characterize the environmental selections on the traits, we applied PCA to the SET matrix and produced a biplot representing loadings of the traits, scores of the edges of the admixture graph, and the loadings of the environmental variables (subsection 2.2).

**FIGURE 1:**
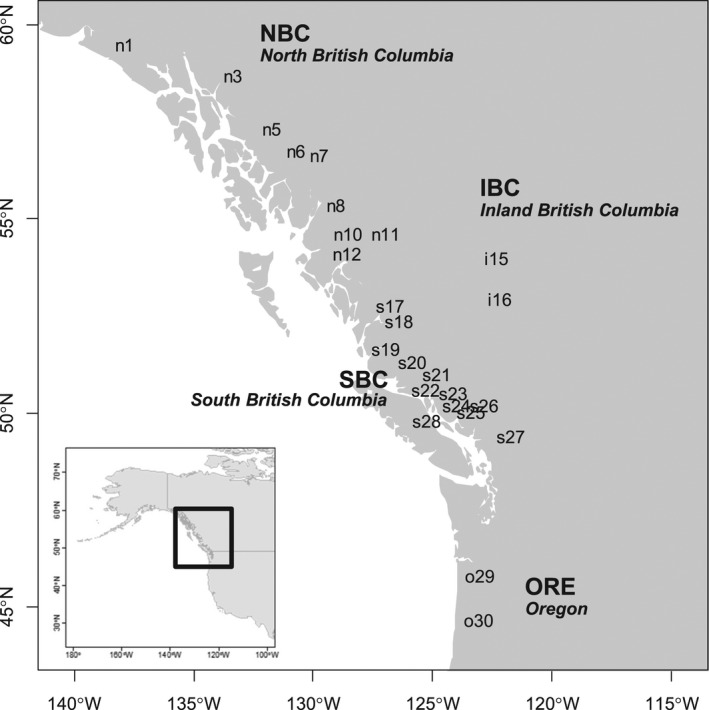
Natural populations of black cottonwood in North America. The sampled individuals were classified into 25 populations. Populations were grouped into the regions: northern British Columbia (NBC), southern British Columbia (SBC), inland British Columbia (IBC), and Oregon (ORE). The populations were labeled by the first characters of the region names and their ID numbers. Data are from Geraldes et al. (2013), McKown, Guy, Klápště, et al. (2014) and McKown, Guy, Quamme, et al. (2014)

We calculated the averages of the environmental values at the sampling locations and phenotypic values of the individuals, and we considered them representative values of the environment and traits, respectively, for each population. We plotted the longitudes and latitudes for the individuals on the map (Figure 1). Because our major concern was identifying correlations between among‐population differentiations of genes, traits, and environmental factors, we selected the SNP with the highest global *F*_ST_ value over 25 populations from each of the 3516 gene regions. Here, we focused on 45 trait variables (Table S1; McKown, Guy, Klápště, et al., 2014; McKown, Guy, Quamme, et al., 2014), namely, adaxial stomata density (ADd), abaxial stomata density (ABd), average of two measurements of leaf rust disease morbidity (DP), 14 phenology traits, 12 biomass traits, and 16 ecophysiology traits (see Table S1). Each sampling location of a population was described by nine environmental/geographical variables: altitude (ALT), longest yearly day length (photoperiod) (DAY), frost‐free days (FFD), mean annual temperature (MAT), mean warmest month temperature (MWMT), mean annual precipitation (MAP), mean summer precipitation (MSP), annual heat–moisture index (AHM, ~MAT/MAP, an indicator of drought), and summer heat–moisture index (SHM, ~MWMT/MSP). The day length and temperature have a north–south cline, whereas temperature, rainfall, and drought have an east–west (coastal to inland) cline (Geraldes et al., 2014). In addition, 18 soil conditions, including the ratio of clay, silt, sand, and gravel, soil depth, bulk density, cation exchange capacity, organic carbon, and pH, each of which were observed in topsoil and subsoil, were obtained from the Unified North American Soil Map (Liu et al., 2013) and used as environmental values of the sampling locations (see Table S2).

## 3 RESULTS

### 3.1 Analysis of simulated data

**FIGURE 2:**
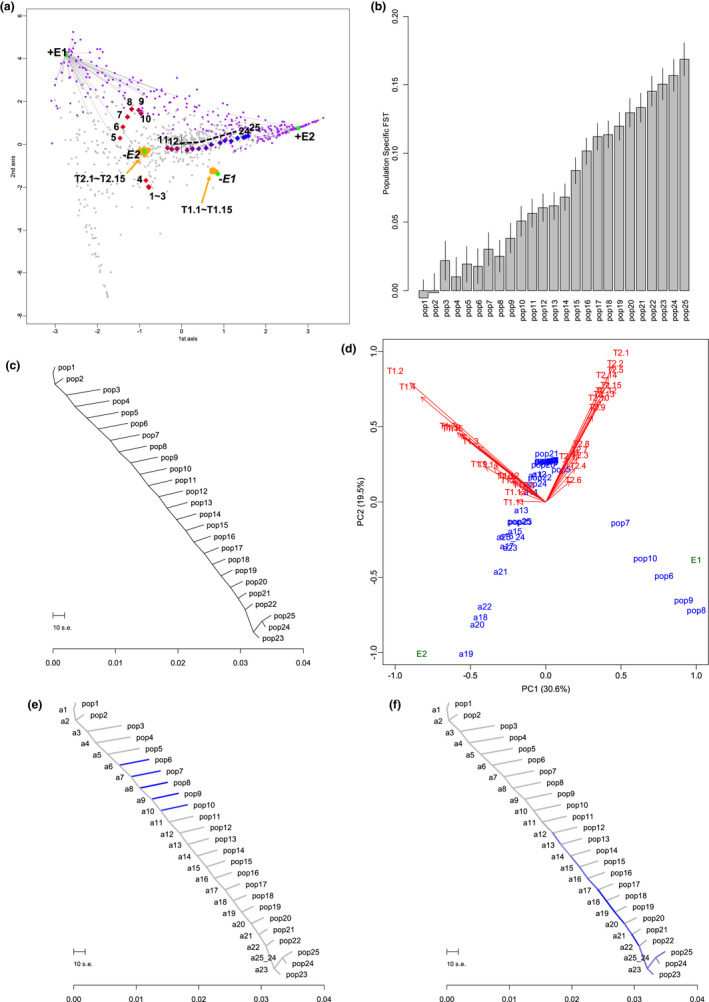
Performance of the exploratory data analysis using simulated data. (a) Correspondence analysis of simulated colonization and adaptation. Green nodes represent environmental factor E. The plus sign (+E) indicates the original environmental value, whereas the minus sign (−*E*) represents the sign‐reversed environmental value. Gray nodes are neutral and environmentally adaptive loci. The orange nodes are the 30 observed traits T1.1,⋯,T1.15 and T2.1.,⋯,T2.15. Each population label is colored by its population‐specific *F*_ST_ value. Numbers from 1 to 25 represent populations, and the color gradients on population labels represent the standardized magnitude of a population‐specific *F*_ST_ value at the sampling point, with colors between blue (for the largest *F*_ST_, which represents the youngest population) and red (smallest *F*_ST_, which represents the oldest population). (b) The estimated population‐specific *F*_ST_ values are shown in the inset. The order of the population‐specific *F*_ST_ estimates was stable in 100 simulations, and the point estimates from the first run were plotted with their asymptotic standard errors. (c) Admixture graph generated by TreeMix. The ancestral nodes of Pop1, …, Pop25 are labeled as a1, …, a25, respectively. (d) PCA of the SET matrix. (e) Colored admixture graph showing the estimated edges of adaptation to environment E_1_. Blue indicates decrease of trait values. (f) Colored admixture graph showing the estimated edges of adaptation to environment E_2_

Figure 2a shows the plot of correspondence analysis. Consistent with the scenario of one‐dimensional stepping‐stone simulation, the locations of the populations were of a curvilinear one‐dimensional pattern. The population‐specific *F*_ST_ values described the chronological order of Pop1 to Pop25 (Figure 2a,b). In our preceding paper, we simulated neutral loci for two‐dimensional stepping‐stone population expansion and confirmed the consistent population structure and population‐specific *F*_ST_ values (Kitada et al., 2021). The environmental factors and environmentally adaptive derived alleles showed positive correlations (connection between green node +E and purple nodes). Consistent with the scenario of $\gamma_{E}\leq 0$ (Appendix S3), the environmental factors (E_1_ and E_2_) and the traits showed negative correlation (connection between green nodes ‐E_1_ and ‐E_2_ and the sets of orange nodes, T1.1, …, T1.15 and T2.1, …, T2.15, respectively). Consistent with the environmental values of the populations, six populations, Pop5 to Pop10, were located near E_1_. Pop15 to Pop25 were located near E_2_ in order.

Consistent with the simulation scenario, the admixture graph created by TreeMix included no admixture events and produced a stair‐stepped tree arraying the 25 populations in order (Figure 2c). PCA of the SET matrix clearly reproduced the history of adaptations to the two types of environments (E_1_ and E_2_) (Figure 2d). Responding to the elevated value of the environment E_1_, the populations along the terminal edges leading to Pop6–Pop10 increased the frequencies of the derived SNP alleles controlling traits T1.1–T1.15 (Figure 2e). Note that derived alleles had negative values for effect size ($\gamma_{G}\leq 0$, Appendix S3). Responding to the linearly increasing value of the environment E_2_, the populations increased the frequencies of the derived SNP alleles controlling traits T2.1–T2.15 during the period of range expansion from Pop15 to Pop25 with population bottlenecks (edges a15–a25, Figure 2f). Note that the analyses of the individual traits partially reconstructed the pattern of the environmental selection and did not provide a solid support for the correct pattern (Figure S2).

### 3.2 Analysis of natural populations of black cottonwood data

#### 3.2.1 Correspondence analysis

**FIGURE 3:**
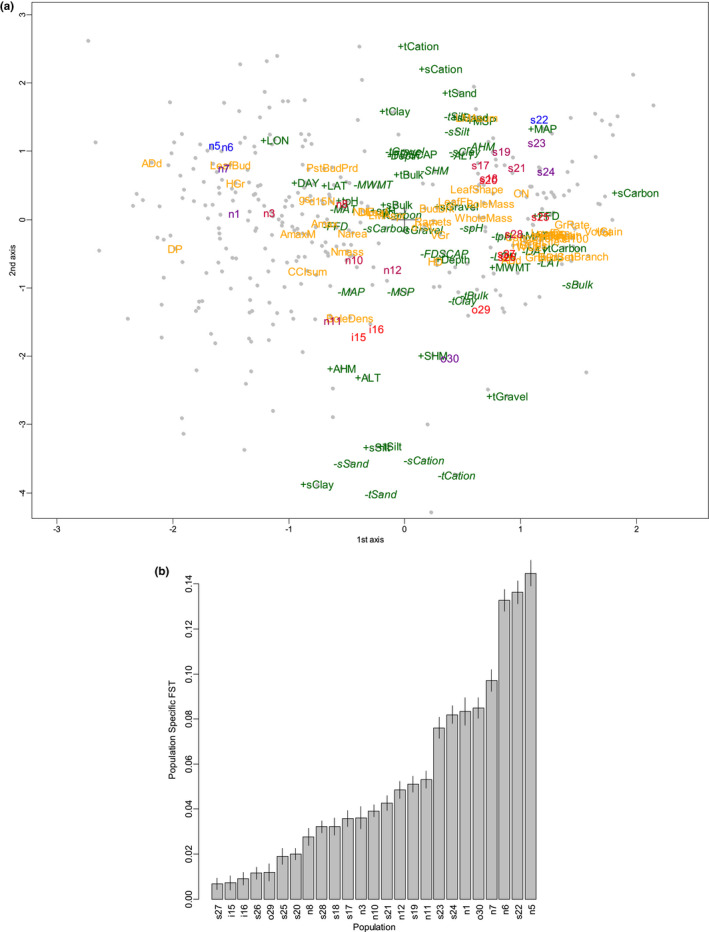
Perspective of the genes, environments, and traits and the history of the natural populations of black cottonwood in western North America. (a) Correspondence analysis. Populations are indicated in the cloud of genes (SNPs, marked as gray dots), environments (green), and traits (yellow). Environment labels with plus signs represent the original environmental values, whereas environment labels with minus signs (in italic) represent the sign‐reversed environmental values. The colors of the populations represent low (red) and high (blue) population‐specific *F*_ST_ values. Labels of traits and environments are described in Tables S1 and S2. (b) Population‐specific *F*_ST_ values of the 25 populations. The population prefixes “n,” “s,” “i,” and “o” represent populations in the regions of northern British Columbia, southern British Columbia, inland British Columbia, and Oregon, respectively. Tables S1 and S2 list the 45 trait variables and 29 environmental variables shown in the figure

The plot of correspondence and correlation analysis provided a sketch of the global structure of genetic differentiation and adaptation (Figure 3a). The colors of the populations, which represented the population‐specific *F*_ST_ values (Figure 3b), indicated distribution expansion in three directions; that is, the expansion from inland (s27, i15, i16) to the coast (s20–s26) and to northern (n1–n12) and southern areas (o29 and o30) (Figures 1 and 3a,b). Population s27, which had the lowest population‐specific *F*_ST_ values, may be better labeled as “si27” because it is located inland.

The color gradient of population‐specific *F*_ST_ values on the population labels indicates that the ancestral population might inhabit the inland area (SBC s27, and IBC i15 and i16). s27 is characterized by warm temperature (MWMT), whereas i15 and i16 are characterized by high altitude (+ALT) and dry conditions year‐round (+AHM, −*MAP*), as shown in the center of Figure 3a. Warm temperatures in summer (+MWMT) were correlated with ARF6 (controlling stamen elongation; Cardarelli & Ghelli, 2020) and flower maturation (Nagpal et al., 2005) (see Appendix S1, Table S3). Dry conditions (+AHM) were correlated with genes associated with drought and osmotic regulation: CBF4 (response to drought and cold stress; Haake et al., 2002; Hussain et al., 2018), XERICO (response to osmotic stress, response to salt stress; Ko et al., 2006), SAL1 (response to water deprivation and salt stress; Wilson et al., 2009), MYB85 (cell wall biogenesis responding water deprivation and salinity; Winter et al., 2007), and APX1 (water deficit; Zandalinas et al., 2016). This indicates that poplar might have initially adapted to the inland area.

Slightly larger population‐specific *F*_ST_ values than those of IBC (Figure 3a,b) indicated that the population expansion might have occurred to the coastal area (SBC), which is characterized by relatively short day length in summer (−*DAY*); this means that the seasonal variation of day length is small in the southern area, with mild temperatures (+MAT, +FFD) and wet conditions year‐round (−*AHM*, +MAP), as plotted in Figure 3a (lower left). The small seasonal variation of day length (−*DAY*) was correlated with abaxial stomata density, which indicated that strong southern sunlight stimulates photosynthesis and requires many stomata. Mild temperatures (+MAT, +FFD) were correlated with genes associated with growth traits: GH3.9 (root growth; Khan & Stone, 2007), GSL12 (signaling during growth and development; Yadav et al., 2014), and iqd2 (leaf growth regulator; Nikonorova et al., 2018). The year‐round wet environment (−*AHM*) was correlated with a gene related to water conditions, HRA1 (response to hypoxia, Giuntoli et al., 2014). The results indicated that populations in SBC were adapted to warmth and oxygen deprivation due to excessive water. After adaptation in SBC, natural populations of black cottonwood might have expanded to the southern area (ORE), which is characterized by warm and dry conditions, particularly in the summer (+SHM). Dry summer (+SHM) was correlated with stress response in the gene NAC090 (salt and drought tolerance; Zang et al., 2019), which revealed that the population adapted to hot and dry summer conditions. In such dry environments, particularly in ORE, the soil consisted of subsoil clay and top soil gravel with little sand to maintain water retention and root growth. On the contrary, the top soil consisted of soil, sand, and clay with little subsoil silt in SBC to control excessive water and maintain good drainage to prevent root rot with cation absorption (tCation and sCation).

Populations in NBC had large population‐specific *F*_ST_ values and little genetic diversity, suggesting that they are young and that natural populations of black cottonwood expanded to the northern area. This area is characterized by long day length in summer (+DAY), which means that day length varies greatly from season to season, and low temperatures (−*MAT*, −*MWMT*, and −*FFD*), as described in Figure 3a. These variables were correlated with adaxial stomata density and leaf rust disease (DP). This finding supports the preceding knowledge that the adaxial stomata compensates for reduced photosynthetic efficiency in the northern area; however, there is a risk of pathogen invasion (Melotto et al., 2006). DAY was correlated with genes associated with light conditions: ACT7 (response to light stimulus; McDowell et al., 1996), PRR7 (circadian rhythm; Alabadí et al., 2001), PRR5 (response to long day conditions; Nakamichi et al., 2005), and GA3OX1 (response to red light and gibberellin; Nelson et al., 2010). These results indicated that the population adapted to the light conditions, which vary greatly among seasons.

#### 3.2.2 PCA and multitrait coadaptation mapped on the admixture graph

**FIGURE 4:**
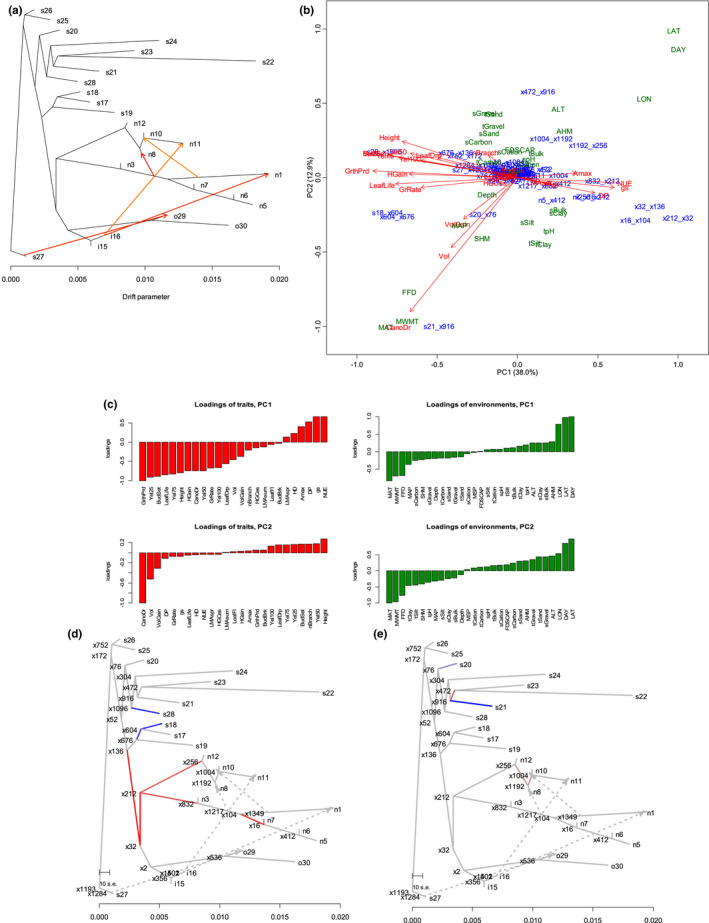
Multitrait coadaptations in the natural populations of black cottonwood in western North America. (a) The admixture graph estimated by TreeMix. Besides the terminal nodes representing the populations, the internal nodes were labeled to enable interpretation of the biplot of PCA. (b) PCA of the SET matrix (Table S5). For each trait, the vector of positive selection parameters that characterize the predicted increase/decrease of the trait value on the admixture graph was estimated using PolyGraph. Edges were expressed by their connecting node pairs. To understand the coadaptation with reference to the local environments, the loadings of the environments were calculated as the sum of products of loadings of the traits and the among‐population correlations between the traits and environments (see Materials and Methods). The labels colored blue (populations) and green (environments) represent edges of the admixture graph and environments. The labels colored red represent traits. Labels of traits and environments are explained in Tables S1 and S2. (c) Loadings of the trait and environmental variables. (d) and (e) Positive selections of the first and second principal component traits. For each principal component, the scores of the edges were obtained as a linear combination of the trait‐specific selection parameter values with the weight of the factor loadings and mapped on the admixture graph. Red/blue colors represent increased/decreased selection, respectively, on principal component trait values

When we set the population i15 as a root, admixture events into populations i15 and i16 were identified from population n13 in NBC (Figure S3). This suggested that the small population‐specific *F*_ST_ values and high genetic diversity observed in i15 and i16 were due to admixture. Therefore, population s27 was tentatively set as a root in the following analysis. We estimated the history of population splits and admixture by applying TreeMix to the population‐specific genotype frequencies of the 34,131 SNPs. The populations expanded their distribution to the coastal region (s20–s26) and extended the range to northward (s17–19). Some extended further northward (n1–n12) and others extended southward (o29, o30) and toward inland (i15, i16) (Figures 1 and 4a). Figure 4a indicated admixtures from the inland populations to the northern populations and between northern populations.

Out of the 45 measured traits, significant associations with genes were detected for 25 traits. For each of the 25 traits, we applied PolyGraph to the populations’ allele frequencies of GWAS‐identified SNPs (see Appendix S2, Table S4) and mapped the positive selection parameters on the admixture graph (Figure S4, Table S5). To further investigate the history of multitrait coadaptation inferred by correspondence analysis, we performed PCA of the SET matrix and obtained the biplot of edges of the admixture graph and trait variables. The factor loadings of the environmental variables were calculated from the factor loadings of the trait variables and the trait–environment correlations (Table S6) and were overlaid on the biplot.

In Figure 4b, edges were expressed by their connecting node pairs of the admixture graph (Figure 4a). The first PC identified the increasing values of the traits related to photosynthesis efficiency, Amax, gs, NUE, and decreasing the length of growing periods, GrthPrd, Yel25, BudSet, LeafLife, Yel75, Height, and HGain, during the populations’ northward extension (Figure 4b,c). The increased efficiency of photosynthesis was accompanied by increased risk of pathogen infection. This was because increased photosynthesis efficiency was caused by the increase of stomatal density that in turn increased the risk of pathogen infection (DP) (McKown, Guy, Quamme, et al., 2014). Reaching the coastal region, the growing season became longer for populations s18, s21, and s28 (Figures 1, 4b–d, Table S1). The second PC described the coadaptation of canopy duration and volume (CanopyDr, Vol, and VolGain), which occurred in the coastal populations s21 and s20 (Figure 4b,c,e).

North–south geographical locations (LAT and LON) and the associated climatic gradient of temperature and length of daylight (MAT, MWMT, FFD, and DAY) were the major environmental factors that drove both first and second PC multitrait coadaptation (Figure 4b,c). It was the soil characters that differentiated the first and second PC coadaptation as the underground environmental constraints (Bingham, 2001). The soil of the first PC included bulk and clay at higher latitudes with a cold and dry climate, and organic carbon and gravel at lower latitudes with a warm climate and more precipitation. However, the soil of the second PC was sand, gravel, and organic carbon at higher latitudes, and clay and silt at lower latitudes (Figure 4c, right).

## 4 DISCUSSION

A population evolves in space and time and responds to variable environments. From its birth, a population may continuously change its distribution range, and the initial localities may have occasionally been exposed to unprecedented environmental stress. In these localities, individuals and populations can acclimate to such environmental stresses by phenotypic plasticity in a short amount of time, and the populations can adapt by changing their geographical distribution or genomes over a long amount of time. We focused on the latter and aimed at understanding the whole history of the populations by conducting an exploratory analysis of multitrait coadaptations. The whole scheme of the analysis has multiple layers. In the first layer, we generated the features that are used as inputs for the second layer of analysis. The features are population‐wide SNP site frequency spectra, genome‐wide association with traits and environments, and polygenic adaptations mapped on the admixture graph. These features were obtained by ever‐evolving population genetic and quantitative genetic procedures. The exploratory analysis is the second layer of analysis incorporating these features.

To reveal the whole scenario of historical change of geographical distribution and adaptation to the new environments, we conducted correspondence analysis that visualizes the population structure in relation with the SNPs, environmental variables, and trait variables. From the biplot, the history of range expansion and differentiation was inferred by the population‐specific *F*_ST_ values. To interpret the adaptations, we referred to the first layer analysis of an association study by searching for the genes that are associated with the environmental variables surrounding the characteristic groups of populations.

To understand the multitrait coadaptation, we analyzed the correlations among the estimated history of positive selections that increased or decreased the trait values. The estimates of the selections were obtained by the first layer analysis in which the admixture graph was constructed and represented the history of population differentiation and admixture; then, the positive selection parameters were mapped on the admixture graph based on the spatial allele frequencies of the SNPs associated with the traits.

We aimed at showing, through the numerical simulation and analysis of empirical data, that the complexity of populations’ life history can be interpreted well solely by integrating the information of among‐population genetic difference and genome‐wide association with multiple environments and multiple traits. A multilayered approach may be a practical choice. However, our approach is still in its infancy. One direction of future study is to include latent variables that are interpreted as key elements of environmental selection and adaptation. Another direction is quantifying the pattern of adaptation. A natural framework is a Bayesian approach that uses the features provided by the first layer analysis as the prior information.

As a final remark, we note that our analysis uses populations as units of analysis. However, populations are often defined by poststratification of the sample. In the case of natural populations of black cottonwood data, we adopted the population assignment provided by the original dataset. The power of our exploratory approach depends on the accuracy of the assignment of the individuals to local populations. Consequently, individual‐level analysis also deserves consideration.

## CONFLICT OF INTEREST

The authors declare that they have no conflict of interest.

## AUTHOR CONTRIBUTIONS

**Reiichiro Nakamichi:** Data curation (lead); Formal analysis (lead); Methodology (supporting); Software (lead); Writing – original draft (equal). **Shuichi Kitada:** Conceptualization (supporting); Investigation (lead); Supervision (supporting); Writing – original draft (equal). **Hirohisa Kishino:** Conceptualization (lead); Funding acquisition (lead); Methodology (lead); Project administration (lead); Supervision (lead); Writing – original draft (equal).

## Supporting information

Fig S1‐S5

Table S1‐S6

Appendix S1‐S3

## Data Availability

The authors affirm that all data necessary for confirming the conclusions of the article are present within the article, figures, and supplementary information. The R codes to perform our representation method and simulations of population colonization are available in Dryad: https://doi.org/10.5061/dryad.wstqjq2p3.
