# Supplementary material for: Exploratory analysis of multi‐trait coadaptations in light of population history
Source: Ecol Evol. 2022 Mar 18;12(3):e8755. doi: 10.1002/ece3.8755 (PMC8933610; doi:10.1002/ece3.8755)
Supplement: Supplementary file 1 — Fig S1‐S5 [file ECE3-12-e8755-s001.pdf]

Supplementary Information for

Exploratory analysis of multi-trait coadaptations in the light of population history

Reiichiro Nakamichi, Shuichi Kitada, and Hirohisa Kishino

This pdf file includes:
Supplementary Figures S1–S5
Supplementary Tables S1–S6 are presented as an Excel file.

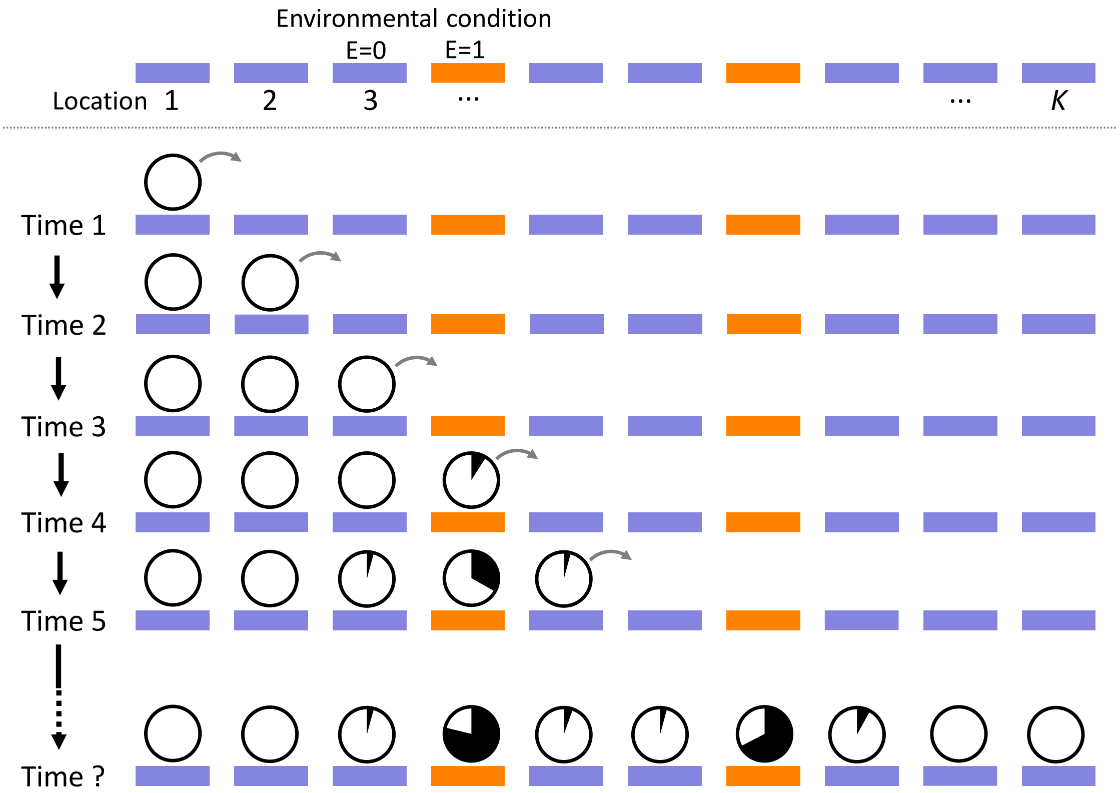

Figure S1 Population expansion and environmental adaptation. Each population has an environmental condition of E=0 (blue) to E=1 (orange). The population colonization started from population 1 and expanded to population *K*. Pie charts denote frequencies of ancestral (white) and derived (black) alleles. Populations were successively colonized every 10 generations, and 1% of N_e individuals migrated to adjacent vacant locations, as indicated by the arrows. Migrated individuals increased to N_e in one generation, and thereafter underwent genetic drift.

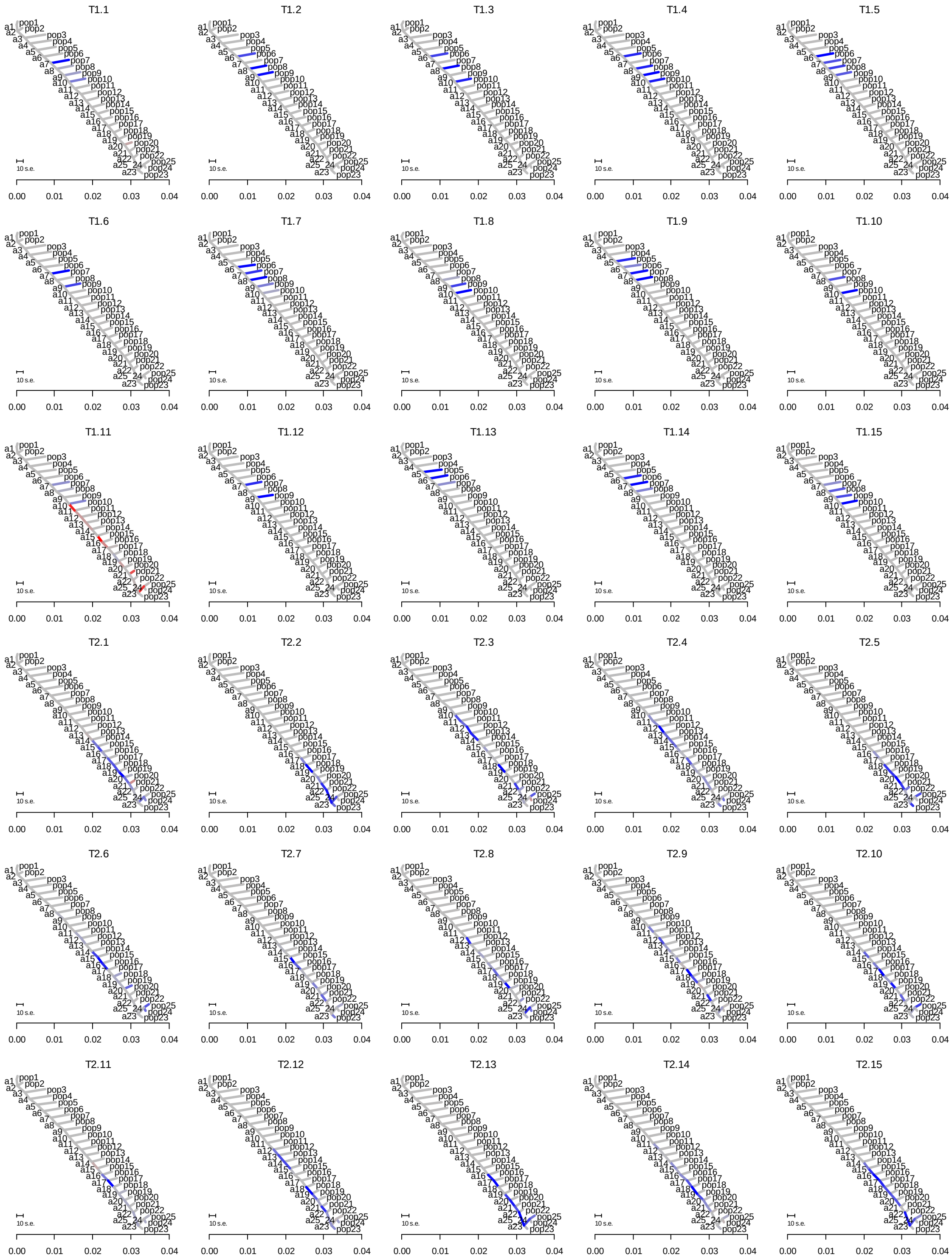

Figure S2 Positive selection parameters of the simulated 30 traits, T1.1, …, T1.15 and T2.1, …, T2.15, mapped on admixture graphs. For each trait, the vector of positive selection parameters that characterize the predicted increase or decrease of the trait value on the admixture graph was estimated using PolyGraph and mapped on the admixture graphs estimated by TreeMix. Red/blue colors represent selection toward increase/decrease of the trait values, respectively.

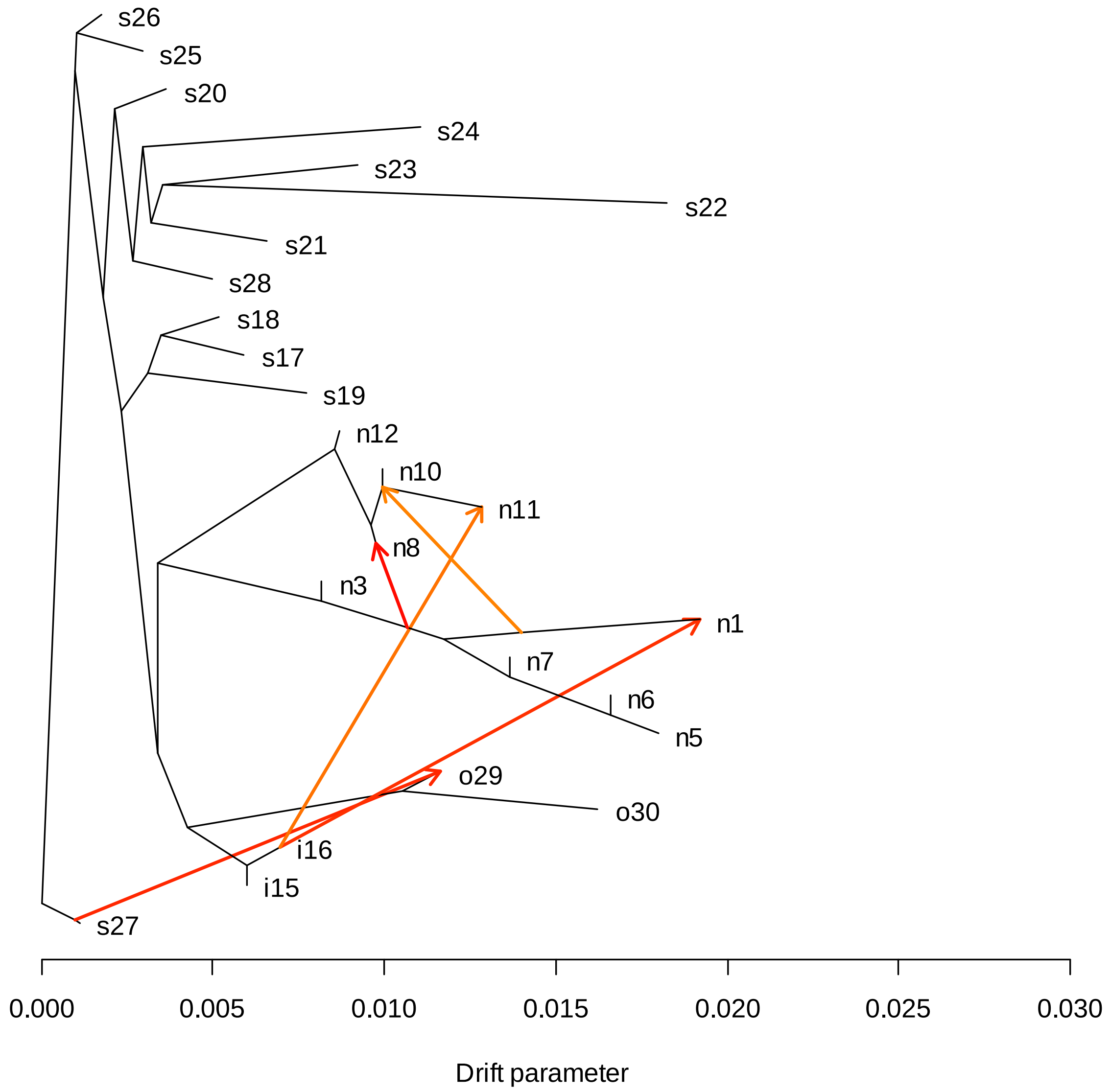

Figure S3 Admixture graph of the western North American wild poplar populations estimated by TreeMix. The root population was assumed to be s27. The graph includes five migration events, some of which represent “time-reversed” admixtures of diverged populations.

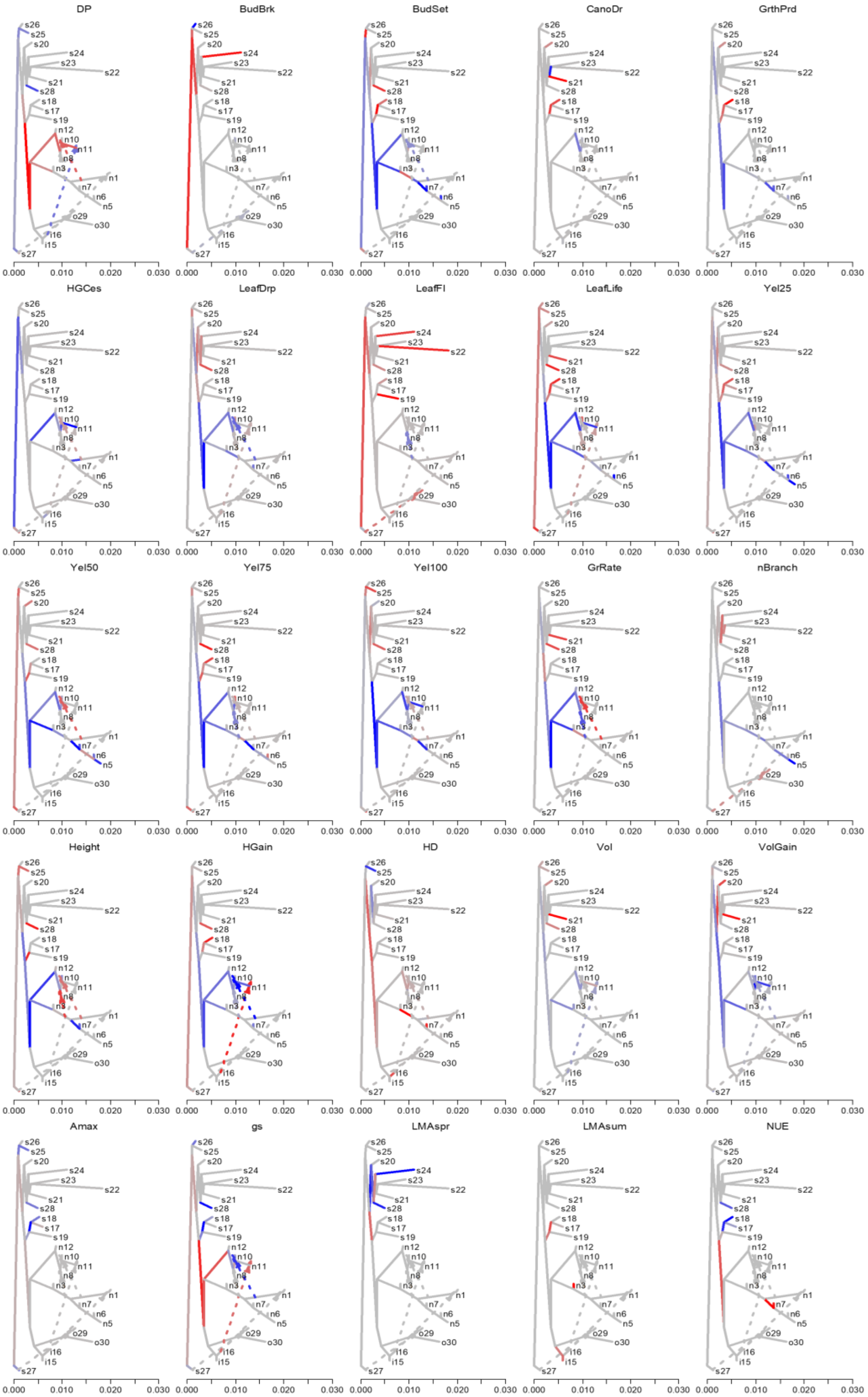

Figure S4 Positive selection parameters of 25 traits mapped on admixture graphs. For each trait, the vector of positive selection parameters that characterize the predicted increase or decrease of the trait value on the admixture graph was estimated using PolyGraph and mapped on the admixture graphs estimated by TreeMix. Red/blue colors represent selection toward increase/decrease of the trait values, respectively.

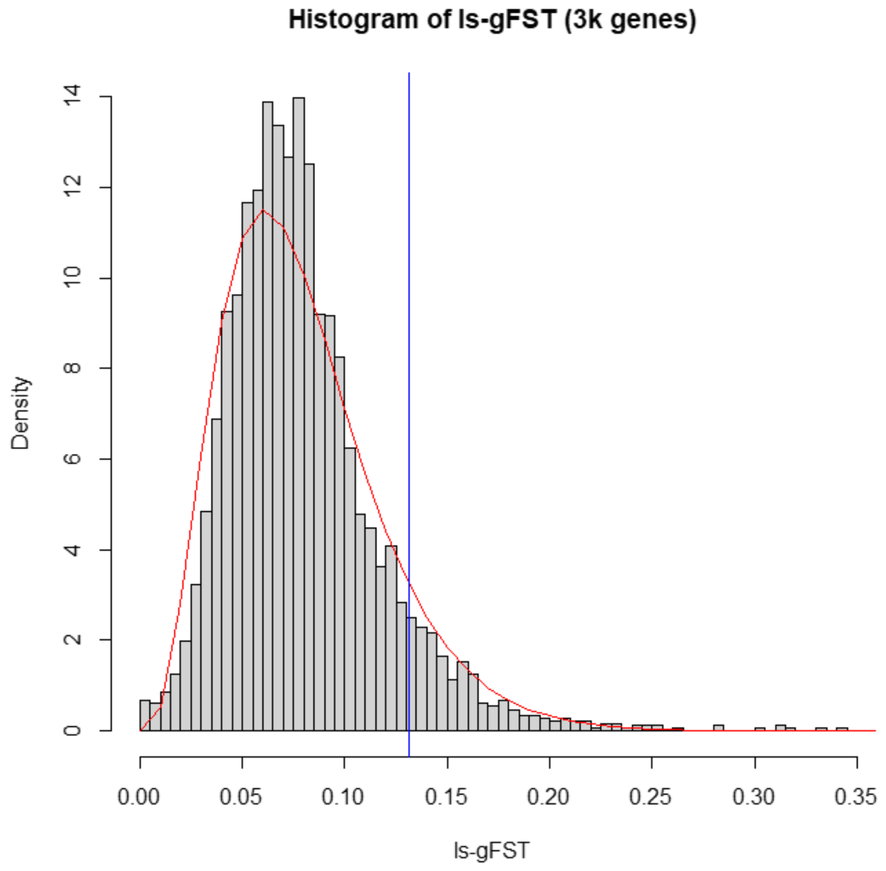

Figure S5 Distribution of the locus-specific global F_{ST} values and fitted gamma distribution. The gray histogram represents the distribution of locus-specific global F_{ST} values. The red line is the fitted gamma distribution. The blue line is the threshold for the upper p-value of 0.1 of the gamma distribution. SNPs on the right side of the blue line were used as the adaptive data set for association analysis.
