# Supplementary material for: Exploratory analysis of multi‐trait coadaptations in light of population history
Source: Ecol Evol. 2022 Mar 18;12(3):e8755. doi: 10.1002/ece3.8755 (PMC8933610; doi:10.1002/ece3.8755)
Supplement: Supplementary file 3 — Appendix S1‐S3 [file ECE3-12-e8755-s003.docx]

**Appendix S1| Gene–environment correlation**

To interpret the adaptation to each local environment type, we identified the significant correlations between the environment and genes (SNPs). We accounted for the correlation structure of the residuals. At each locus, the variance matrix of the observed allele frequencies reflects the genetic drift and gene flow and the sampling variance (Nicholson, Smith, Jónsson, Gústafsson, & Stefánsson, 2002; Coop, Witonsky, Di Rienzo, & Pritchard, 2010). Here, we adopted the frequentist approach to choose significant pairs given a value of false discovery rate (FDR) (1% for the simulation and 5% for the poplar data).

We consider $K$ populations derived from a common ancestral population and $L$ loci of biallelic neutral markers. Let $p_{i}^{l}$ and $p_{A}^{l}$ be the derived allele frequency of marker $l$ ($l=1 - L$) in population $i$ ($i=1 - K$) and the (unobserved) ancestral population. Given the samples from the populations, the allele frequencies are estimated by the observed counts as $\hat{p}_{i}^{l}={n_{i}^{l}}/{n_{i}}$, where $n_{i}$ is the number of the samples (twice of the number of individuals) in population $i$, and $n_{i}^{l}$ is the count of derived allele at marker $l$ in population $i$.

The among-population mean allele frequencies vary largely among neutral loci. Therefore, we incorporate the contribution of variable allele frequencies in the ancestral population to estimate the among-population correlation that is shared among loci. Given the allele frequency in the ancestral population, the variance–covariance matrix of the allele frequencies $\boldsymbol{p}^{l}=\left( {p_{1}^{l},\ldots,p}_{K}^{l} \right)^{'}$ at locus $l$ is formulated as

$$\mathbf{E}\left( \boldsymbol{p}^{l} \right)=p_{A}^{l}\mathbf{1}$$

$\mathbf{V}\left( \boldsymbol{p}^{l} \right)=p_{A}^{l}\left( 1-p_{A}^{l} \right)\boldsymbol{\Omega}$ **.**

$\nu_{ij}=\boldsymbol{\Omega}_{ij} \left( i, j=1, \ldots, K \right)$represents the among-population covariance (Weir & Hill, 2002; Coop, Witonsky, Di Rienzo, & Pritchard, 2010). The variance and covariance of the observed allele frequencies are

$V\left( \hat{p}_{i}^{l} \right)=V\left( p_{i}^{l} \right)+E\left[ p_{i}^{l}(1-p_{i}^{l})/n_{i} \right]$

$$=p_{A}^{l}\left( 1-p_{A}^{l} \right)\nu_{ii}+\frac{1}{n_{i}}\left( p_{A}^{l}-\left( p_{A}^{l}\left( 1-p_{A}^{l} \right)\nu_{ii}+{p_{A}^{l}}^{2} \right) \right)$$

$$=p_{A}^{l}\left( 1-p_{A}^{l} \right)\left( \left( 1-\frac{1}{n_{i}} \right)\nu_{ii}+\frac{1}{n_{i}} \right)$$

$\mathrm{COV}\left( \hat{p}_{i}^{l},\hat{p}_{j}^{l} \right)=COV\left( p_{i}^{l},p_{i}^{l} \right)=p_{A}^{l}\left( 1-p_{A}^{l} \right)\nu_{ij} \left( i\neq j \right)$.

From this, we obtained the moment estimator $\boldsymbol{\Omega}$ as

$$\hat{\nu}_{ii}=\frac{1}{L}\sum_{l=1}^{L} \begin{aligned} \left( \frac{\frac{{\hat{p}_{i}^{l}}^{2}}{{\hat{\bar{p}}}_{.}^{l}\left( 1-{\hat{\bar{p}}}_{.}^{l} \right)}-\frac{1}{n_{i}}}{1-\frac{1}{n_{i}}} \right) \\ \end{aligned}$$

$$\hat{\nu}_{ij}=\frac{1}{L}\sum_{l=1}^{L} \left( \frac{\hat{p}_{i}^{l}\hat{p}_{j}^{l}}{{\hat{\bar{p}}}_{.}^{l}\left( 1-{\hat{\bar{p}}}_{.}^{l} \right)} \right) \left( i\neq j \right),$$

where ${\hat{\bar{p}}}_{.}^{l}={n^{l}}/n$. Because most SNPs rarely have alleles in equilibrium (Wright, 1931), these estimates are accurate when many neutral loci are available. With this estimated variance–covariance matrix of the allele frequencies, we obtained the variance–covariance matrix of the observed counts, ${\hat{\boldsymbol{p}}}^{l},$ as

$\hat{V}\left( \hat{p}_{i}^{l} \right)={\hat{\bar{p}}}_{.}^{l}\left( 1-{\hat{\bar{p}}}_{.}^{l} \right)\hat{\nu}_{ii}+{{\hat{\bar{p}}}_{.}^{l}\left( 1-{\hat{\bar{p}}}_{.}^{l} \right)}/{n_{i}}$

$\hat{\mathrm{COV}}\left( \hat{p}_{i}^{l},\hat{p}_{j}^{l} \right)={\hat{\bar{p}}}_{.}^{l}\left( 1-{\hat{\bar{p}}}_{.}^{l} \right)\hat{\nu}_{ij}$.

Assuming the normality of the estimated regression coefficient, the p-value was calculated by contrasting the coefficient with the standard error based on the standard generalized least squares method. Out of environment–gene (SNP) pairs and environment–trait pairs, we selected the significant pairs with an FDR of $0.05$ using the Benjamini–Hochberg procedure (Benjamini & Hochberg, 1995).

To improve the power of detecting associations between genes and environments, we focused on the SNPs that were over-differentiated among populations compared with the level of differentiation of neutral loci. First, we obtained the maximum likelihood estimates of the locus-specific global *F*_ST_ values (Beaumont & Bolding, 2004) using R package FinePop2 in CRAN. We fitted a gamma distribution to the distribution of these locus-specific global *F*_ST_ values by maximum likelihood procedure using fitdistr function in MASS package. The *F*_ST_ values were far below 1, at least in the simulation and real-data analyses (see Supplementary Figure S5). We assumed that most of the SNPs were neutral, and that the fitted distribution approximates the distribution of locus-specific global *F*_ST_ values of neutral sites. As a set of over-differentiated SNPs, we collected the SNPs with *F*_ST_ values with upper p-value $<0.1$ in this gamma distribution.

**Appendix S2| Kernel-based GWAS and estimation of the effects**

To make the dataset for PolyGraph analysis, we conducted GWAS for each of the traits using genome-wide gene-based analysis by considering genes as test units (Deng et al. 2020). For a given gene, a joint effect of multiple SNPs within the gene is obtained by Gaussian kernel function. Association between a trait and the candidate kernel function of a gene is evaluated by the generalized association test based on *U*-statistics, which use environmental factors as the fixed effects to control the correlation structure of the populations. Genes that have significant association with the trait were selected by 5% FDR. We adopted these genes as the explanatory variables that explain the population adaptation in the PolyGraph model. Finally, for each trait, we performed simple linear regression on each of the SNPs on the significant genes for the trait, and estimated regression coefficient of each gene. Then, we adopted the sign (+1 or −1) of the coefficient as the selective pressure in the PolyGraph analysis. For the analysis of poplar, we performed this association test between 45 traits and 3,516 genes, and obtained 22 traits that had significant genes (see Supplementary Table S3).

**Appendix S3| Simulation of range expansion and adaptation: environmental selection and fitness of derived alleles contributing to the latent traits**

The genotype $G=0, 1, 2$ of the environmental adaptation locus contributes to a latent trait $T\left( G,E \right)$ with the interaction of genotype $G$ and environmental factor $E$:

$T(G,E)=\gamma_{G}G+\gamma_{E}E+\gamma_{GE}G\times E+\varepsilon_{T}$ $\varepsilon_{T}\sim N(0,1)$. (A1)

The survival probability $S(T)$ of the trait value $T$ is described as the probability that $T$ is positive:

$S(T)=\mathrm{Prob}(T>0)$. (A2)

The larger the trait, the greater the chance of survival. The survival probability of a genotype $G$ under environmental condition $E$, $S(G|E)$, is given as $S\left( G | E \right)=S(T(G,E))$. In population $i$, given the frequency $P_{G}^{(t)}$ of genotype $G$ at generation $t$, the allele frequency at the next generation is obtained as$P_{G}^{(t+1)}={P_{G}^{\left( t \right)}S(G|E_{i})}/{\sum_{G} \left\{ P_{G}^{\left( t \right)}S(G|E_{i}) \right\}}$. We note that only the relative values of survival probabilities are relevant for the population genetic dynamics.

The derived allele is assumed to be advantageous over the ancestral allele under the environmental condition $E=1$, whereas it is disadvantageous under the normal environmental condition $E=0$. Therefore, we consider the case where $\gamma_{G}\leq0$, $\gamma_{E}\leq0,$ and $\gamma_{GE}\geq0$. We set simulation parameters $r$ and $s$ to control environmental effect $\gamma_{E}$, genetic effect $\gamma_{G}$, and gene–environment interaction $\gamma_{GE}$. The parameter $r \left( 0<r<1 \right)$ was introduced to represent the stress of environmental condition $E=1$ and was defined by the ratio of the survival probabilities between the two conditions:

$r={S\left( G=0|E=1 \right)}/{S\left( G=0|E=0 \right)}$. (A3)

The parameter $s$ $\left( \geq0 \right)$ represents the fitness of the derived allele in the environmental condition $E=1$:

$1+s={S\left( G=2|E=1 \right)}/{S\left( G=0|E=1 \right)}$. (A4)

We assumed the cost of adaptation by reversing the fitness in the normal environmental condition $E=0$:

${S\left( G=2|E=0 \right)}/{S\left( G=0|E=0 \right)}=\frac{1}{1+s}$ (A5)

The coefficients $\gamma_{E}$, $\gamma_{G}$, and $\gamma_{GE}$ were obtained from $r$ and $s$. First, we noted that, from equations (A1) and (A2), $S\left( G=0|E=0 \right)=\frac{1}{2}$ and $S\left( G=0|E=1 \right)=\Phi\left( \gamma_{E} \right)$. $\Phi$ is the cumulative distribution of the standard normal distribution. Hence, from equation (A3), we obtained $\gamma_{E}=\Phi^{-1}\left( \frac{r}{2} \right)$. Similarly, we obtained $\gamma_{G}={\frac{1}{2}\Phi}^{-1}\left( \frac{1}{2\left( 1+s \right)} \right)$ from equation (A5). Finally, we obtained $\gamma_{GE}=\frac{1}{2}\left( \Phi^{-1}\left( \frac{rs}{2} \right)-2\gamma_{G}-\gamma_{E} \right)$ from equation (A4).

References:

1. Beaumont, M. A., & Balding, D. J. (2004). Identifying adaptive genetic divergence among populations from genome scans. Molecular Ecology , 13, 969–980. <https://doi.org/10.1111/j.1365-294X.2004.02125.x>
2. Benjamini, Y., & Hochberg, Y. (1995). Controlling the false discovery rate: A practical and powerful approach to multiple testing. Journal of the Royal Statistical Society: Series B (Statistical Methodology) , 57, 289–300. <https://doi.org/10.1111/j.2517-6161.1995.tb02031.x>
3. Deng, Y., He, T., Fang, R., Li, S., Cao, H., & Cui, Y. (2020). Genome-wide gene-based multi-trait analysis. Frontiers in Genetics , 11, 437. <https://doi.org/10.3389/fgene.2020.00437>
4. Nicholson, G., Smith, A. V., Jónsson, F., Gústafsson, O., Stefánsson, K., & Donnelly, P. (2002). Assessing population differentiation and isolation from single-nucleotide polymorphism data. Journal of the Royal Statistical Society: Series B (Statistical Methodology) , 64, 695–715. <https://doi.org/10.1111/1467-9868.00357>
5. Weir, B. S., & Hill, W. G. (2002). Estimating F-statistics. Annual Review of Genetics , 36, 721–750. <https://doi.org/10.1146/annurev.genet.36.050802.093940>
